# Research on the Technologies of Food Extraction, Pressing and Extrusion

**DOI:** 10.3390/foods13233721

**Published:** 2024-11-21

**Authors:** Christianne E. C. Rodrigues, Maria Carolina Capellini, Daniel Gonçalves

**Affiliations:** 1 Laboratório de Engenharia de Separações (LES), Departamento de Engenharia de Alimentos (ZEA), Universidade de São Paulo (USP), P.O. Box 23, Pirassununga 13635-900, Brazil; maria.capellini@alumni.usp.br; 2 Laboratory of Food Technology (LTA), Center for Agricultural Sciences and Technologies (CCTA), Universidade Estadual do Norte Fluminense Darcy Ribeiro (UENF), Campos dos Goytacazes, Rio de Janeiro 28013-602, Brazil; danielg@uenf.br

This Editorial refers to the Special Issue “Research on the Technologies of Food Extraction, Pressing and Extrusion”, which highlights new opportunities and challenges in advancing the development of new food products and increasing processing efficiency. The processing of foods, along with the assertive use of agroindustrial byproducts and wastes from vegetable and fruit handling, has the potential to mitigate hunger, food insecurity, and malnutrition. Various technologies have been proposed to enhance the extraction of macro and minor constituents from foods, wastes, and byproducts. These technologies include pressing, extrusion, drying, and the association of these methods with traditional or modern solvent extraction techniques.

This Special Issue of *Foods* focuses on studies that could offer solutions to (i) maximize the recovery yields of lipids, proteins, fibers, vitamins, or antioxidant compounds; (ii) improve the availability of macro and minor nutrients; (iii) preserve the nutritional value of processed foods through mechanical approaches, such as extrusion and pressing; and (iv) modify structural, rheological, functional, or sensory aspects to increase acceptability and commercial applications. The keywords for this Special Issue are food processing; green extraction; extrusion; pressing; and drying.

Five high-quality manuscripts were accepted for publication and are included in this Special Issue. The papers underwent a rigorous review procedure concentrated on food processing and relevant analysis in the context of extraction, pressing, drying, and extrusion. 

The main topics evaluated in the articles of this Special Issue of *Foods* include drying, oil extraction by cold pressing, solvent extraction, supercritical carbon dioxide extraction, and the extrusion of protein materials. All evaluated processes, materials, and parameters are summarized in Table 1.

Yue et al. [1] conducted a comprehensive study on the effects of several drying methods, including rotary microwave vacuum (RMVD), radio frequency vacuum (RFVD), vacuum far-infrared (VFID), vacuum (VD), hot air (HD), and natural drying (ND) on *Codonopsis pilosula* slices. The objective was to evaluate how these methods influence the drying efficiency and quality of the final product. Key variables such as drying time, effective moisture diffusivity, and the retention of bioactive compounds such as flavonoids, lobetyolin, and syringin were analyzed. RMVD proved to be the fastest method, completing the process in 18 min, while VD required the longest time of 1220 min. Among the methods, RMVD was the most effective in preserving bioactive components and displayed the highest moisture diffusivity. Based on these findings, the authors recommended RMVD for industrial applications, emphasizing the need for further process optimization to maximize product quality.

Grajzer et al. [2] accomplished a comparative analysis of oil extraction from wild strawberry (*Fragaria vesca* L.) seeds using two methods: cold pressing and supercritical carbon dioxide (SCO_2_) extraction. The study aimed to evaluate the effectiveness of these techniques in recovering valuable bioactive compounds, such as fatty acids, phytosterols, tocopherols, and carotenoids. It was observed that SCO_2_ extraction led to oils being enriched in phytosterols and tocopherols, while cold pressing was more effective in preserving carotenoids and squalene. Furthermore, the duration of SCO_2_ extraction played a crucial role in determining the concentration of certain compounds, with shorter extraction periods yielding higher levels of tocopherols and polyphenols. In conclusion, both extraction methods were deemed acceptable for valorizing wild strawberry seeds in various industries, including food and biomedical sectors, contributing to the circular economy. Nonetheless, additional cytotoxicity tests are recommended to confirm the safe application of the recovered oils.

Aussanasuwannakul et al. [3] investigated the valorization of soybean residue (okara) by extracting its oil with SCO_2_. The oil extraction was conducted with and without ethanol as a cosolvent, attaining a recovered oil yield of 18.5% after 450 min. The study aimed to evaluate the physicochemical and functional properties of the defatted and full-fat powders obtained through this process. Significant variations were observed in appearance, color, and composition between the two powder types. The SCO_2_ extraction enhanced the defatted powder’s water absorption and oil-binding capacity, although its swelling capacity was reduced. Adding ethanol as a cosolvent increased the extracted oil’s total phenolic content, isoflavones, and antioxidant capacity. Additionally, the defatted okara powder exhibited superior gelation and water absorption properties when compared with the full-fat powder. The authors concluded that the oil and defatted powder have promising potential as food ingredients. However, further studies on sensory characteristics and process optimization are necessary to enhance the yield and concentration of bioactive compounds.

Magalhães et al. [4] explored the employment of ethanol as an alternative and greener solvent than hexane for oil extraction from peanut press cake. The study examined variables such as temperature, solid-to-solvent mass ratio, and the number of contact stages in a sequential cross-current extraction configuration. These factors were analyzed in relation to the performance of the extraction and the properties of the defatted solids. Hexane achieved an oil extraction yield of 86 ± 2% in two stages at 55 °C with a solid-to-solvent ratio of 1:4, while ethanol required a higher temperature (75 °C), a larger solvent amount (solid-to-solvent ratio of 1:5), and three contact stages to reach a comparable oil yield of 87 ± 4%. The defatted solids produced from both methods had high protein content (>45 mass%) and a high nitrogen solubility index (>80%), making them suitable as potential material to produce protein concentrates or isolates. The oils extracted with both solvents had the same fatty acid composition of the cold-pressed peanut oil, although the ethanol-extracted oil showed a higher free acidity and a more intense color. As a renewable solvent, the authors concluded that ethanol could replace hexane for extracting residual oil from peanut press cake, contributing to a more sustainable production process.

Pennells et al. [5] examined how dry heat pretreatment influences the functionality of soy, chickpea, and pea proteins when used to produce texturized vegetable protein (TVP) through low-moisture extrusion. The study focused on the effects of varying pretreatment temperatures on critical properties, such as pasting behavior, water absorption capacity, and changes in the density and color of the extrudates. The results showed that the optimal pretreatment temperature was 120 °C, which enhanced the pasting properties and maintained water absorption capacity. However, at higher temperatures, such as 160 °C, a significant reduction in functionality was observed, with the extrudates becoming denser and darker. This behavior indicates that the transformation of proteins through denaturation, unfolding, or aggregation significantly influences the ultimate properties of the extruded materials. The researchers concluded that the precise control of pretreatment conditions is critical for optimizing protein-based meat analogs. However, additional sensory evaluations are necessary to ensure that these products meet consumer expectations in terms of taste and texture.

In conclusion, the studies contained in this Special Issue significantly contribute to advancing food processing technologies, particularly in drying, pressing, extraction, and extrusion processes. Each study emphasizes the optimization of process parameters to balance efficiency and sustainability while preserving valuable nutrients and functional properties. Additionally, the research underscores the potential of these technologies to support the circular economy by enabling the reintegration of byproducts into food and biomedical applications. Future research should focus on refining these techniques, with a particular emphasis on sensory evaluation, safety assessments, and the scalability of production processes, to enhance their commercial viability and environmental benefits.

## Figures and Tables

**Table 1:** Published contributions in the Special Issue.

Authors/Reference	Evaluated Processes	Materials	Evaluated Variables
Yue et al. [1]	Rotary microwave vacuum drying	*Codonopsis pilosula* slices	Color
	Radio frequency vacuum drying		Effective moisture diffusivity
	Vacuum far infrared drying		Drying time (drying kinetics)
	Vacuum drying		Active ingredients retainment (total flavonoids, lobetyolin, and syringin)
	Hot air drying		Microstructure (porosity)
	Natural drying		Model fitting
Grajzer et al. [2]	Oil cold pressingOil extraction with supercritical carbon dioxide	Wild strawberry (*Fragaria vesca* L.) seed	Extraction time
			Composition (tocopherol, total polyphenol, carotenoids, squalene, phytosterol)
			Overall quality
			Cytotoxicity
			Oxidative stability
Aussanasuwannakul et al. [3]	Oil extraction with supercritical carbon dioxide (with and without ethanol)	Soybean residue (okara)Okara oilDefatted okara powder	Kinetics of extraction
			Chemical composition (soluble dietary fiber, protein, fatty acid profile, phenolic and aglycone contents)
			Physicochemical
			Functional properties (water and oil absorption capacities, swelling capacity)
			Particle size
			Health-promoting properties
			Antioxidant capacity
			Consistency
Magalhães et al. [4]	Oil extraction with hexane and ethanol	Peanut press cake	Temperature
			Solid/solvent ratio
			Number of contact stages in a cross-current extraction
			Oil extraction yield
			Fatty acid composition
			Physical properties (density, viscosity)
			Color
			Free acidity
			Protein content
Pennells et al. [5]	Dry heat pretreatment and low moisture extrusion	Soy, chickpea, and pea protein	Pretreatment temperature
			Water absorption and water-holding capacity
			Color
			Pasting properties
			Particle size distribution
			Thermal analysis (DSC)
			Physical properties (viscosity, density)
			Textural properties
			Empirical regressions and Monte Carlo simulation
